# Identification of leptospiral 3-hydroxyacyl-CoA dehydrogenase released in the urine of infected hamsters

**DOI:** 10.1186/1471-2180-14-132

**Published:** 2014-05-21

**Authors:** Takaya Segawa, Kazuko H Nomura, Sharon Yvette Angelina M Villanueva, Mitsumasa Saito, Kazuya Nomura, Nina G Gloriani, Shin-ichi Yoshida

**Affiliations:** 1 Department of Bacteriology, Graduate School of Medical Sciences, Kyushu University, Fukuoka 812-8582, Japan; 2 Department of Biological Sciences, Graduate School of Sciences, Kyushu University, Fukuoka 812-8582, Japan; 3 Department of Medical Microbiology, College of Public Health, University of the Philippines-Manila, Manila 1000, Philippines

**Keywords:** Leptospirosis, 3-hydroxyacyl-CoA dehydrogenase, Urine, Hamster, Diagnosis

## Abstract

**Background:**

Leptospirosis is a global zoonosis caused by pathogenic *Leptospira*. The non-specific clinical signs and symptoms of leptospirosis lead to its misdiagnosis. To date, there is still no reliable rapid test kit that can accurately diagnose leptospirosis at bedside or in field. In this research, with the ultimate goal of formulating a rapid and accurate diagnostic tool for leptospirosis, we aimed to identify leptospiral proteins excreted in urine of infected hamsters, which are thought to mimic Weil’s disease.

**Results:**

Hamsters were subcutaneously infected with leptospires, and the general attributes of urine as well as the proteins excreted in it were examined. Some leptospiral proteins were found to be excreted in the urine from the early phase of infection. The most important finding of this study was the detection of the lipid-metabolizing enzyme, 3-hydroxyacyl-CoA dehydrogenase (HADH), before the onset of illness, when leptospires were not yet detected in the urine of infected hamsters.

**Conclusions:**

This is the first report on the detection of leptospiral HADH in the host urine, which may be a possible candidate leptospiral antigen that can be used in the early diagnosis of human and animal leptospirosis.

## Background

Leptospirosis is a global zoonosis caused by the pathogenic *Leptospira* spp. Outbreaks of leptospirosis usually occur after heavy rains followed by floods in tropical and subtropical developing countries, and recreational activities in developed countries [1,2]. The genus *Leptospira* is comprised of 21 species and more than 300 serovars. Animals may become maintenance hosts of some serovars or incidental hosts of others [3]. Infection of accidental hosts may cause severe or fatal disease. Wild rats, dogs, buffaloes, horses, and pigs are known to contract the disease and the surviving animals maintain the organisms in their kidneys. Infected animal urine contains leptospires, which may contaminate the environment once excreted, becoming a new source of infection for humans and susceptible animals. Infection of humans or animals occurs when leptospires penetrate both normal and injured skin and mucosal surfaces after direct contact with the urine of infected animals or indirectly from contaminated environments [1,4].

Signs and symptoms of human leptospirosis are usually mild, however, 5% of cases develop the severe form presenting jaundice, renal failure, and pulmonary hemorrhage [1,2,4-6]. This zoonotic infection is treatable but its early phase has clinical presentations similar to many other diseases thereby complicating its clinical diagnosis. Early diagnosis of leptospirosis is essential to prevent progression to the severe stage because antibiotic treatment is effective when it is initiated early in the course of the disease.

The gold standards for diagnosis of leptospirosis are isolation of *Leptospira* by culture from blood, urine or tissues of infected hosts and the microscopic agglutination test (MAT) to detect antibody. However, results of these diagnostic methods can only be evaluated more than 10 days after the onset of illness. Furthermore, technical expertise is needed in order to perform the culture and MAT. In attempts to replace these two methods, other diagnostic methods were developed such as enzyme-linked immunosorbent assay (ELISA) [7], polymerase chain reaction (PCR) [8-11], and so on [12-16]. However, these are not simple or rapid tests that can be used at bedside [1,2,4,17] and sophisticated equipment is needed in order to perform PCR. In addition, with the exception of PCR, the sensitivities of the other assays are not satisfactory, especially during the acute phase of infection [18]. At present there is a lack of available kits that are able to detect leptospiral antigens in patient samples such as urine. Furthermore, there is also a need for simple and rapid leptospirosis diagnostic kits that are cheap, highly sensitive, highly specific, and can easily be used at bedside or in the field.

Urine is expected to be the best sample for diagnosis, because the urine of leptospirosis patients contains leptospiral antigens and can be collected easily [19-26]. Urine of patients has often been used for culture of *Leptospira*, however, more information on proteins can be obtained from urine [27].

The golden Syrian hamster is susceptible to *Leptospira* infection, and acute leptospirosis in the hamster model reproduces the severe form of human leptospirosis, and is therefore useful in evaluating diagnostic methods [28]. In this study, we analyzed the characteristics and protein components of *Leptospira*-infected hamster urine in order to identify proteins that may be possibly used in developing rapid and accurate leptospiral antigen diagnostic kits. We identified a leptospiral protein, 3-hydroxyacyl-CoA dehydrogenase (HADH), which was found to be excreted in the urine of hamsters during the early phase of infection.

## Results

### Changes in urine characteristics of hamsters during *Leptospira* infection

**Figure 1:**
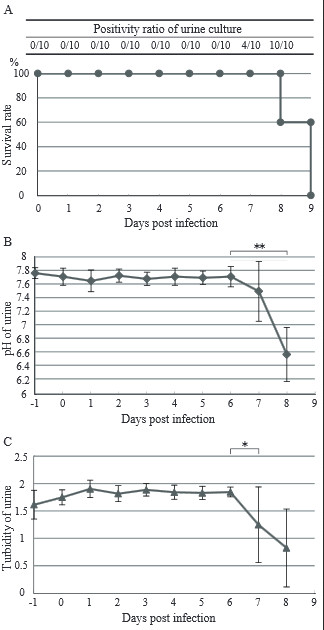
**Survival of infected hamsters and sequential change of general urinary conditions during *Leptospira *infection. (A)** Survival rate of infected hamsters and *Leptospira*-positivity ratio of the urine culture were checked every day. Hamsters were infected with 10^3^ leptospires and urine was collected every day from pre-infection to just before death. Chemical analysis of hamster urine was done using urinalysis paper and absorbance was also measured at 600 nm. Infected hamsters became moribund from the eighth day post infection. Leptospires were recovered from the urine from the seventh day after infection. Three independent experiments were done (n = 10) and the sum of the survival rate of the 10 hamsters are shown. **(B and C)** Urinary pH **(B)** and absorbance **(C)** changed after the seventh day. Hamster infection experiments were repeated three times. The sum of the turbidity and pH values were taken and the average (±SD) are shown (n = 10). *: p < 0.05, **: p < 0.01.

Hamsters were subcutaneously infected with 10^3^ leptospires (strain K64), and their urine was collected daily in metabolic chambers for 6 h. All infected hamsters became markedly sick after the seventh day showing decreased mobility and body weight, ruffled fur, and decreased food and water intake, and became moribund from the eighth day post infection (Figure 1A). We confirmed that the cause of death was leptospirosis because leptospires were isolated from the blood, urine, and organs (lungs, livers, kidneys, spleens, and brains) of moribund hamsters. Normal hamster urine was alkaline (Figure 1B) and milky (Figure 1C). However, it became acidic (Figure 1B) and clear (Figure 1C) after the seventh day of infection. Urine culture was negative for leptospires until the sixth day, but became positive from the seventh day post infection (Figure 1A). Using urinalysis strips, we also found that the levels of glucose, specific gravity, blood, protein and bilirubin increased at the same time, whereas the levels of urobilinogen, nitrite, leukocyte and ketone did not change. Urinary protein level was 30 mg/dl before infection, and increased to 300 mg/dl on the seventh day post infection.

### Changes in hamster urinary proteins during leptospiral infection

**Figure 2:**
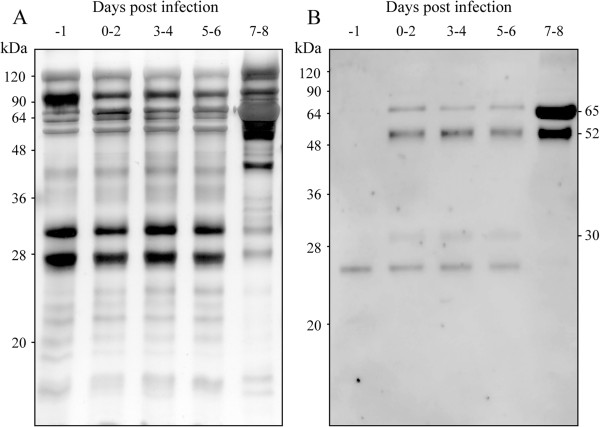
**SDS-PAGE and immunoblotting of hamster urine protein during *Leptospira *infection. (A)** Compositions of hamster urinary proteins were compared according to infection periods. Hamster urine was collected and prepared for SDS-PAGE. The urine of three hamsters was mixed for each infection period. The protein content of each sample was 5 μg. After separation with SDS-PAGE, the gel was stained by silver staining. **(B)** The anti-*L. interrogans* pAb recognized leptospiral proteins in infected-hamster urine by immunoblotting. These experiments were repeated three times, and the representative data are shown in this figure.

The hamster urine was collected daily from pre-infection to just before death and the protein compositions were compared using SDS-PAGE. Until the sixth day post infection, urinary protein compositions were almost the same as that of pre-infection. Significant change was observed after 7 days of infection, particularly an increase in the density of approximately 66 kDa protein which is thought to be albumin (Figure 2A).

For detecting leptospiral proteins in hamster urine, we performed immunoblotting with rabbit polyclonal antibody against *L. interrogans* serovar Manilae. Three bands with sizes of 65, 52, and 30 kDa were detected in the post-infection urine (Figure 2B). These bands were already detected during the early phase of post-infection. During this phase, the hamster appeared healthy and no viable leptospires were recovered from the urine. 26 kDa protein was detected in urine before and during infection so this protein was not a result of infection.

### Comparative analysis of urinary protein composition before and after *Leptospira* infection by using two dimensional electrophoresis (2-DE) and immunoblotting

**Figure 3:**
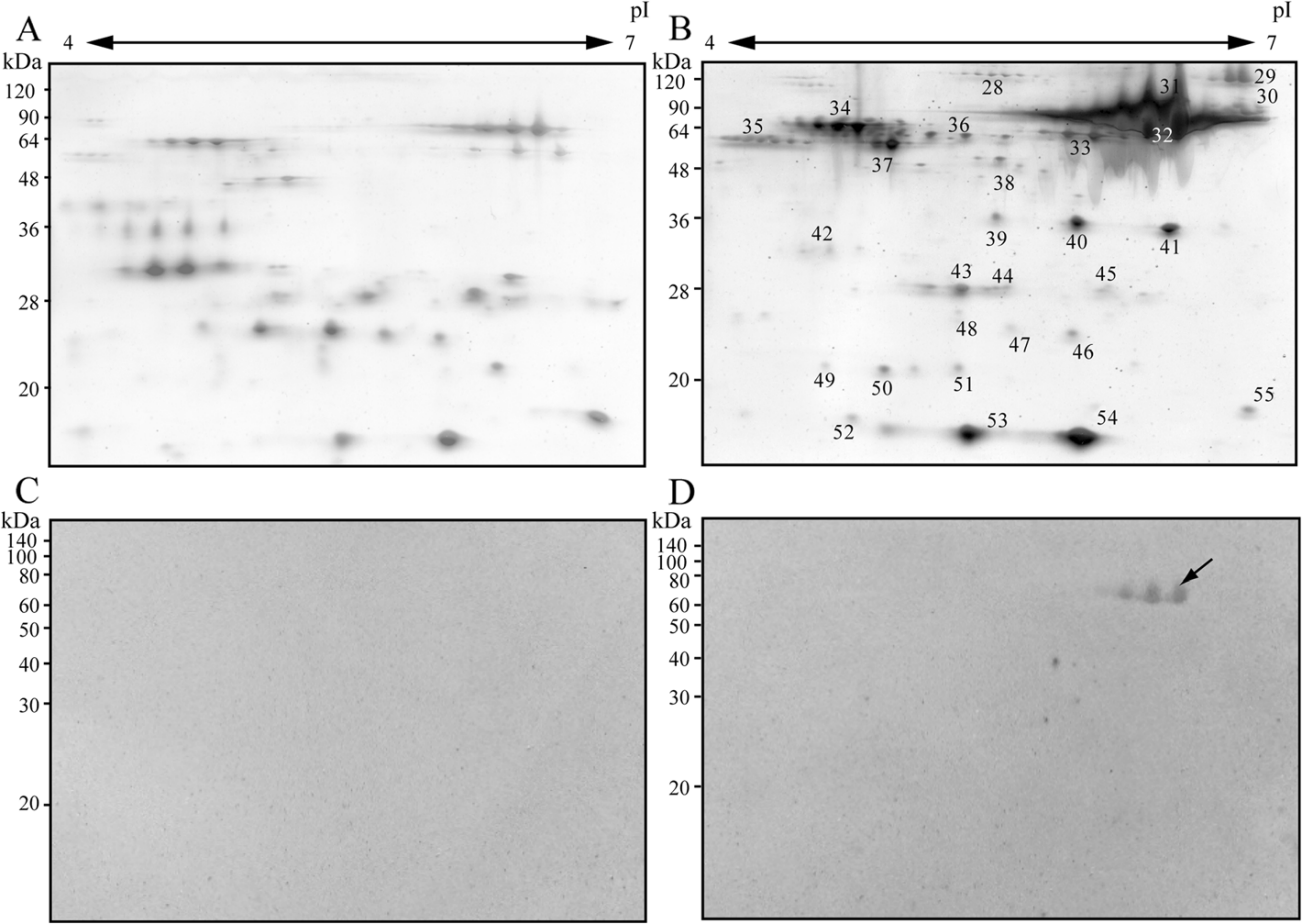
**2-DE analysis of normal and infected hamsters urine.** Urinary protein patterns of hamsters were compared by 2-DE analysis; **(A, C)** before infection; **(B, D)** 7–8 days post-infection. The urine of three hamsters was mixed for each infection period. The total protein content of each sample was 20 μg. Each pattern of urinary protein was separated by pI (4–7), 12.5% acrylamide gel, and subsequently silver staining **(A, B)**, or immunoblotting with anti-*L. interrogans* pAb was done **(C, D)**. Arrows **(D)** show spots of 60 kDa that reacted with the polyclonal antibody at 7–8 days post-infection. Each experiment was repeated three times, and the representative data are shown in this figure.

As shown in the results of SDS-PAGE (Figure 2A), the composition of urinary proteins was found to have changed drastically after the seventh day of infection. To compare these components in detail, the urinary proteins of pre-infection and seventh day of infection were analyzed by 2-DE (Figure 3). The 2-DE pattern of urinary proteins changed after *Leptospira* infection. It was found that the level of approximately 66 kDa protein in the urine significantly increased on the seventh day (Figure 3A and B). By immunoblotting using anti-*L. interrogans* pAb, the 60 kDa spots were detected (Figure 3D, arrow). However, spots with other sizes were not detected in hamster urine (Figure 3D). 2-DE analysis were also done for urine samples at 3–4 days infection however the protein pattern was found to be the same as pre-infection urine samples and further analysis by immunoblot was also unable to detect any protein spots (data not shown).

### Proteins with increased levels after *Leptospira* infection

A total of 29 protein spots that had increased density after infection (Figure 3B) were selected and analyzed by LC/MS/MS analysis. Database analysis showed that these urinary proteins were albumin, alpha-1-antitrypsin, alpha-1-inhibitor III, angiotensinogen, apolipoprotein A-I, ceruloplasmin, haptoglobin, pancreatic trypsin 1, pregnancy protein 60 kDa, protease serine 1, transferrin, transthyretin, AMBP protein, vitamin D-binding protein and Cu/Zn superoxide dismutase (Table 1). Most of these proteins were serum proteins, which are usually detected in the urine of patients with renal failure. It is noteworthy that some of the leptospiral proteins were also identified as ABC transporter, 3-hydroxyacyl-CoA dehydrogenase (HADH), chloride channel, and conserved hypothetical proteins in the urine (Table 2).

**Table 1:** List of hamster proteins excreted in urine that had increased levels of expression during infection

**Spot no.**	**Accession no.†**	**Protein annotation**	**MW (kDa)**	**pI**	**Urinary marker of diseases (Reference)**
**28**	gi:110347564	ceruloplasmin isoform b [*Mus musculus*]	121872	5.53	Acute renal transplant rejection [29,30]
**29**	gi:83816939	alpha-1-inhibitor III [*Rattus norvegicus*]	165038	5.7	No reports
**30, 32, 33, 38**	gi:58585560	albumin [*Microtus fortis fortis*]	70261	5.91	Glomerular disease [31,32], Diabetes mellitus type 2 [33]
**31**	gi:17046471	transferrin [*Mus musculus*]	78794	6.92	Glomerular disease [31,32]
**34**	gi:68052028	Alpha-1-antitrypsin precursor	46019	5.55	Glomerular disease [32]
**35**	gi:191388	pregnancy protein 60 kDa	47574	8.53	No reports
**36**	gi:19705570	angiotensinogen [*Rattus norvegicus*]	52177	5.37	Chronic kidney disease [34]
**37**	gi:193446	vitamin D-binding protein [*Mus musculus*]	54647	5.26	Glomerular disease [31,32]
**39-41**	gi:41019123	Haptoglobin precursor	39090	5.76	Glomerular disease [31,32], Diabetes mellitus type 2 [33]
**42**	gi:2497695	AMBP protein precursor	39669	5.87	Diabetes mellitus type 2 [33,35]
**43-45, 48**	gi:62899898	Apolipoprotein A-I precursor	30720	5.86	Glomerular disease [36]
**46, 51, 52**	gi:6981420	pancreatic trypsin 1 [*Rattus norvegicus*]	26627	4.71	Pancreatitis [31]
**47, 49**	gi:16716569	protease, serine, 1 [*Mus musculus*]	26802	4.75	No reports
**50, 53, 54**	gi:6981684	transthyretin [*Rattus norvegicus*]	15852	5.77	Glomerular disease [31,32], Diabetes mellitus type 2 [33]
**55**	gi:226471	Cu/Zn superoxide dismutase	15923	6.03	Endemic nephropathy [37]

^†^http://www.ncbi.nlm.nih.gov/protein/.

**Table 2:** **List of leptospiral proteins excreted in hamster urine during *Leptospira *** **infection**

**Spot no.**	**Accession no.**^**†**^	Locus tag^*^	**Protein annotation**	**MW (kDa)**	**pI**	**Predicted location**^**#**^
**32**	gi:45599159	LIC10012	conserved hypothetical protein	61792	9.27	Unknown
	gi:45599713	LIC10580	ABC transporter, atp-binding protein	71297	9.3	Cytoplasmic membrane
	gi:45601755	LIC12676	conserved hypothetical protein	76551	5.75	Cytoplasm
	gi:45602095	LIC13023	conserved hypothetical protein	51182	8.23	Cytoplasm
	gi:45602258	LIC13191	conserved hypothetical protein	65453	6.51	Cytoplasm
	gi:45602297	LIC13229	conserved hypothetical protein	68742	9.21	Unknown
	gi:45602365	LIC13300	3-hydroxyacyl-CoA dehydrogenase	47865	8.65	Cytoplasm
	gi:45602427	LIC13362	chloride channel	67352	8.07	Cytoplasmic membrane

^†^http://www.ncbi.nlm.nih.gov/protein/.

^*^http://aeg.lbi.ic.unicamp.br/world/lic/.

^#^The proteins were predicted with PSORTb (http://www.psort.org/psortb/).

### Identification of HADH in hamster urine

**Figure 4:**
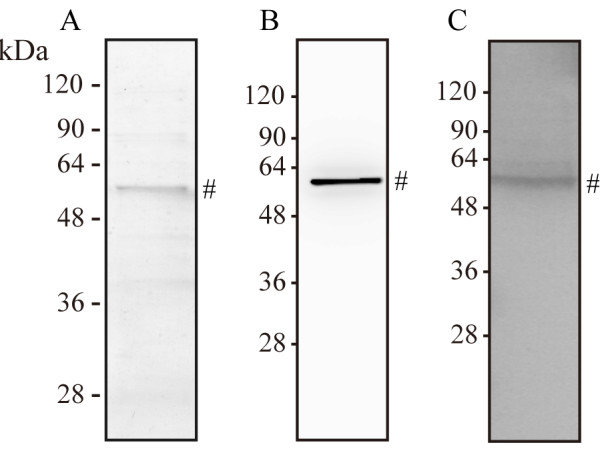
**SDS-PAGE and immunoblotting of recombinant leptospiral HADH. (A)** The rHADH with His-tag was produced by *E. coli* and purified by cobalt resin. In total, 1 μg of the protein was run by SDS-PAGE and CBB staining. **(B)** Anti-His-tag antibody and **(C)** anti-*L. interrogans* pAb detected the protein. Sharp signs indicate recombinant protein bands of 52 kDa. These experiments were repeated three times, and the representative data are shown in this figure.

As mentioned in the previous section, candidate leptospiral proteins in urine were selected based on the results of LC/MS/MS analysis. In order to identify leptospiral proteins that are excreted in hamster urine during infection, recombinant proteins for each selected protein were made. The proteins were screened by immunoblotting with anti-*L. interrogans* pAb. Among them, only HADH reacted to the antibody. The amino acid sequence of HADH are shown in the Additional file 1: Table S1 and had a coverage of 27%. The rHADH was purified with TALON® Metal Affinity Resin (Clontech) and its expression was confirmed with coomassie brilliant blue (CBB) staining (Figure 4A) and immunoblotting by anti-His (C-term) antibody (Figure 4B). The anti-*L. interrogans* pAb also recognized the rHADH (Figure 4C).

### Detection of HADH in infected hamster urine with antiserum

**Figure 5:**
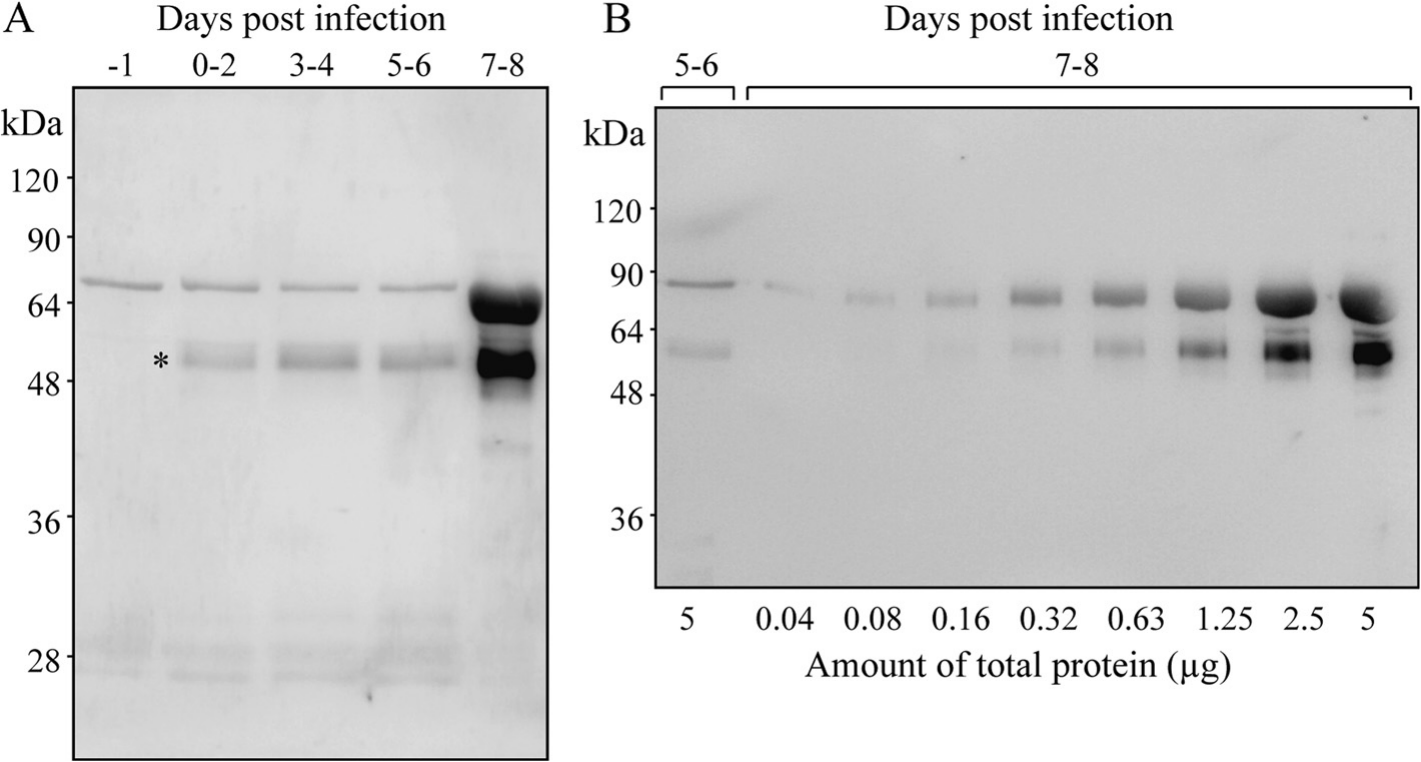
**Immunoblotting of infected hamster urine by anti-HADH antisera. (A)** Rabbit anti-rHADH serum was able to detect the 52 kDa protein (*) in the urine of infected hamsters (n = 3). Each lane contained 5 μg of protein. **(B)** It was revealed, by serial dilution of urine, that HADH increased in the said specimen during the seventh day post infection. These experiments were repeated three times, and the representative data are shown in this figure.

We produced anti-rHADH antiserum in rabbits, and examined its reactivity to rHADH by immunoblotting. The rabbit antiserum recognized the recombinant protein (data not shown). We then performed immunoblotting of urine samples as in Figure 2B and the antiserum reacted with the post-infection samples (Figure 5A). The reacted protein increased after the seventh day of infection (Figure 5B). The protein was found to be excreted in the urine before leptospires were shed (Figure 1A).

## Discussion

We confirmed, by immunoblotting, that several leptospiral proteins were shed in the urine of infected hamsters from the early phase of infection (Figure 2B). On the 7-8^th^ day post-infection, the amount of 52 and 65 kDa leptospiral antigens increased. It was suggested that the proportion of 30 kDa proteins decreased because of rich albumin passing into the urine. Furthermore, we performed 2-DE for a detailed examination of protein components. Patterns of urinary proteins were different between pre-infection and after the seventh day of infection. As mentioned earlier, the infected hamster urine consisted mostly of albumin, consequently we determined proteins that had increased expression. In 2-DE-immunoblotting, 60 kDa proteins were detected by anti-*L. interrogans* pAb (Figure 3D). However, though proteins with 52 and 30 kDa molecular weights were detected in SDS-PAGE-immunoblotting (Figure 2B), they were not found by 2-DE-immunoblotting (Figure 3D). This may be because the two proteins were diluted in 2-DE gel during pI separation or had specific pI outside 4–7. From the amino acid sequence, molecular weight of HADH is 52 kDa and this supports the probability that the 52 kDa band in immunoblotting of urine (Figure 2B), recombinant HADH study (Figure 4), and dilution experiments of urine (Figure 5) is leptospiral HADH. However in 2-DE-immunoblotting analysis, anti-*L. interrogans* pAb detected around 60 kDa protein which is revealed as leptospiral HADH by LC/MS/MS. Molecular weight shift like this (from 52 to 60 kDa) is sometimes observed in these kinds of experiments, and HADH was included in 60 kDa proteins in the 2-DE- immunoblotting (Figure 3D).

The most significant finding in our study was the detection in infected hamster urine of leptospiral protein LIC13300, which is 3-hydroxyacyl-CoA dehydrogenase (HADH) and is one of the intracellular enzyme proteins. This protein is classified as an oxidoreductase in fatty acid metabolic processes. It specifically catalyzes the third step of beta oxidation. Long-chain fatty acids are utilized by *Leptospira* as the sole carbon source and are metabolized by beta-oxidation. Therefore, a large amount of HADH may be produced intracellularly and released to get carbons and energy by oxidizing free fatty acid.

We produced rabbit antiserum against recombinant leptospiral HADH to detect the protein in infected hamster urine. The advantage of using anti-HADH pAb compared to the anti-pathogenic leptospires pAb is that the former is more specific than the latter. Using this antiserum, the protein (52 kDa) was successfully detected in urine after infection (Figure 5A), and an increase in band thickness at 7–8 days post infection was confirmed by serial dilution (Figure 5B). It seems that the appearance of 65 kDa protein in immunoblotting (Figure 5A) was due to non-specific reactions because normal hamster urine had the 65 kDa protein (Figure 5A) and normal rabbit serum also reacted with such protein (data not shown). During 0–6 days after infection, urine still appeared normal and leptospires were not shed in urine. Further study is needed to identify these proteins.

Hamsters and humans also have enzymes similar to leptospiral HADH. The amino acid sequences of this protein are conserved among *Leptospira* spp., however, the amino acid homology between hamster or human and *L. interrogans* serovar Copenhageni were only 25.08% or 32.44%, respectively. It is, therefore, expected that the antisera against leptospiral HADH cannot recognize the protein of hamsters.

Several studies previously reported that the abundant proteins or LPS on the surface of outer membrane were suitable as targets for vaccine and diagnosis of leptospirosis such as outer membrane proteins [38,39], LIC11207 [40], OmpL1 [41,42], MPL17 and MPL21 [43], HbpA [44], LigA [45], LP29 and LP49 [46], LipL32 [47-50], LipL21 [50,51], LipL41 [42], flagellin protein [52]. Moreover, it was also reported that different proteins were expressed in leptospires shed in chronically infected rats compared to leptospires cultured *in vitro*[53], and that the leptospires in rat urine affected urinary protein composition [54]. However, we were not able to identify any of the previously reported leptospiral proteins in the urine either by immunoblotting with anti-*L. interrogans* pAb or MS/MS analysis. The polyclonal antibodies were produced in rabbits, and we confirmed that proteins were recognized by this antibody using immunoblotting and MS/MS analyses. The antibody could recognize some membrane proteins such as LipL32 and LipL41 when bacterial cells were used for immunoblotting (unpublished data). However, leptospiral membrane lipoproteins were not detected in the urine, probably due to their low concentration. These results suggest that not only membrane proteins but also intracellular proteins, such as HADH, can be used as candidates for leptospirosis diagnosis.

We investigated the changes in the attributes of hamster urine prior to infection and a day just before death in a hamster model, and found that the conditions drastically changed one day prior to death. The pH of hamster urine is usually about 8, and it was found to have become acidic before death (Figure 1B). Urinary test results suggest that this acidification was caused by renal failure, like nephritis. Hamster urine is usually cloudy due to a high concentration of calcium carbonate [55]. But, it became clear on the day prior to death due to leptospirosis. Calcium carbonate is deposited in alkali conditions, and dissolved in acidic conditions. *Leptospira-*infected hamster urine was about pH 6, and the urine remained clear even in alkaline condition induced by adding sodium hydroxide. It means that the amount of calcium carbonate excreted in urine decreased just before the hamsters succumbed to infection. After the seventh day of infection, viable leptospires could be recovered from the urine. Thus, it is suggested that leptospires are not shed from the kidneys until just before death.

Most of the urinary proteins detected in this study were associated with host renal failure such as acute renal transplant rejection [29,30], glomerular disease [31,32,36], diabetes mellitus type 2 [33,35], chronic kidney disease [34], pancreatitis [31], and endemic nephropathy [37]. The proteins identified in our study, except for leptospiral HADH, are biomarkers known to be involved in renal failure, but are not specific for *Leptospira* infection. Albumin was the main protein detected in infected hamster urine during the end stage of infection (Figure 2). This is one of the plasma proteins and its primary function is to maintain the colloidal osmotic pressure in both the vascular and extra-vascular spaces. The urine-excreted proteins can serve as markers for glomerular disease [31,32] and diabetes [33].

## Conclusions

HADH was detected in urine before the onset of illness in our hamster model of leptospirosis. This is the first study reporting that leptospiral HADH is released in the urine during the infection. Therefore, this protein could be applicable in early diagnostic assays for human leptospirosis.

## Methods

### Bacteria

*Leptospira interrogans* serovar Manilae strain K64 that was isolated from the kidneys of a rat in the Philippines [56] was used in this study, and cultured in modified Korthof’s medium supplemented with 10% rabbit serum at 30°C. Prior to experiments, strain K64 was passaged through hamsters to maintain its virulence. Strain K64 passaged less than ten times *in vitro* was used for experiments. LD50 of strain K64 was determined by infecting hamsters with serially diluted leptospiral suspension [56,57]. As a result, the LD50 of K64 strain was 10^0^.

### Animals

Male golden Syrian hamsters (Japan SLC, Inc., Shizuoka, Japan), 4 weeks of age, were injected subcutaneously with 10^3^ low-passaged (less than 10× *in vitro*) leptospires at a final volume of 1 ml Korthof’s medium. As negative controls, animals were injected with Korthof’s medium only. The urine of infected animals was collected by housing them in metabolic chambers for 6 hours daily until they were moribund. Hamster kidneys, livers, spleens, lungs and brains were collected aseptically and squeezed into modified Korthof’s medium containing 5-FU using 5 ml syringe, and incubated at 30°C [56]. Five hundred microliters of culture supernatant was sub-cultured into fresh medium without 5-FU the next day and was kept at 30°C and examined for growth of leptospires daily for one month. Moribund hamsters and those that survived infection after 9 days were sacrificed through inhalation with sevoflurane (Maruishi Pharmaceutical, Japan).

All animal experiments were reviewed and approved by the Ethics Committee on Animal Experiment at the Faculty of Medical Sciences, Kyushu University. The experiments were carried out following the Regulations for Animal Experiments of Kyushu University and The Law (No. 105) and Notification (No. 6) of the Government of Japan.

### Urinalysis

The pH of hamster urine was tested using pH test paper BTB (07010060, Advantec, Tokyo, Japan). Glucose, bilirubin, ketone, specific gravity, blood, protein, urobilinogen, nitrite, and leukocyte were measured with N-MULTISTIX® SG-L (Siemens Healthcare Diagnostics Inc., NY). The turbidity of hamster urine was measured using Wallac ARVO sx 1420 multilabel counter (Perkin Elmer, Waltham, MA, USA) at a wavelength of 600 nm.

### Pre-treatment of urine for gel electrophoresis

Due to the small amount of urine collected, urine from three infected hamsters was pooled and used in the experiments. For proteomic analysis, urine samples were first centrifuged at 1500 × g for 10 min at 4°C to remove debris. The supernatants were concentrated and desalted to remove interfering substances by centrifugation at 7500 × g for 30 min at 4°C using a centrifugal filter device (Amicon Ultra 4 molecular mass cutoff, 10-kDa; Merck Millipore, Billerica, MA, USA) as previously described [58]. The desalted concentrates were stored at −20°C until further use. Protein concentration in urine was determined using 2-D Quant Kit (GE Healthcare UK Ltd, Little Chalfont, UK) and processed for gel electrophoresis.

### Sodium dodecyl sulfide–polyacrylamide gel electrophoresis (SDS-PAGE)

For SDS-PAGE, the concentrated and desalted urine samples were dissolved in Laemmli sample buffer (Bio-Rad Laboratories, BioRad, Hercules, CA, USA) with 5% beta-mercaptoethanol and incubated at 94°C for 5 min. SDS-PAGE was performed with 10% acrylamide gels. Electrophoresis was performed using a Mini-PROTEAN tetra cell (Bio-Rad Laboratories, BioRad, Hercules, CA, USA) for 120 min at 20 mA in Tris-glycine running buffer (25 mM Tris, 192 mM glycine, 0.1% sodium dodecyl sulfate). Separated proteins were stained using Silver Stain MS Kit (WAKO, Osaka, Japan).

### Two dimensional electrophoresis (2-DE)

2-DE of the urine samples was analyzed using the Multiphor II Electrophoresis system (GE Healthcare UK Ltd, Little Chalfont, UK) according to the manufacturer’s instructions with some modifications. Briefly, the desalted urine sample was dissolved and recovered with 400 μl of 8 M urea, 4% CHAPS and 50 mM Tris/HCl (pH 8.0). Ten mM DTT and 1% Pharmalyte, broad range pH 3–10 (GE Healthcare UK Ltd, Little Chalfont, UK) including range pH 4–7 were added as rehydration buffer prior to loading for the first dimension. Samples were directly added into the rehydration buffer and the 11 cm immobilized gradient strip (pH 4–7) was allowed to swell overnight at room temperature. The isoelectric focusing (IEF) conditions were as follows: (i) 1 min at a 300 V gradient, (ii) 1.5 h at a 3500 V gradient, (iii) 5 h at 3500 V, with a 50 μA per strip maximum at 15°C. The completed first-dimensional strip was subjected to 2-D SDS-PAGE with 12.5% acrylamide gel. Separated proteins were stained by silver staining as mentioned above.

### Cloning and expression of recombinant HADH

A 1311-bp LIC13300 DNA fragment was amplified using oligomers LIC13300-F 5′-GGAATTCCATATGAGAGAAATCAAAACAGTAACAG-3′ and LIC13300-R 5′-CCGCTCGAGTCCTTTGAAAAGTGAACGAGC-3′ designed based on *L. interrogans* serovar Copenhageni genome sequences (GenBank accession YP_003205). PCR was performed with KOD plus ver. 2 PCR kit (Toyobo, Osaka, Japan) from strain K64. Cycling conditions were: 95°C, 5 min, followed by 40 cycles at 95°C, 1 min, 50°C, 1 min, 68°C, 2 min, and a final extension cycle of 5 min, 68°C. PCR product was digested with *Nde*I and *Xho*I (Roche, Basel, Schweiz), ligated to *Nde*I- and *Xho*I- digested expression vector, pET-28a (+) (Novagen, San Diego, CA). The ligated plasmid was amplified in *E. coli* DH5α and purified using Midi PlusTM Ultrapure Plasmid Extraction System (Viogene, Taipei, Taiwan). After confirming the presence of correct inserts by sequence analysis, the plasmid was transformed in *E. coli* (DE3). Cultures were grown to OD_600_ = 0.5 and protein expression was induced with 1 mM isopropyl-beta-D-thiogalactopyranoside (IPTG), and incubated at 25°C overnight. His-tagged LIC13300 recombinant protein (rHADH) was purified under native conditions with TALON® Metal Affinity Resin (Clontech) as previously described [59].

### Antiserum against rHADH

One female Japanese white rabbit (Biotek. Co.,Ltd., Japan) weighing 1.5 kg was immunized subcutaneously with 30 μg of the recombinant protein. The rHADH was mixed with an equal volume of complete Freund’s adjuvant (Sigma-Aldrich, St. Louis, MO) to make an emulsion. Four subsequent booster injections were given at two-week intervals in the same way, by using incomplete Freund’s adjuvant (Sigma-Aldrich, St. Louis, MO). One week after the final immunization, the blood of rabbit was collected through cardiac puncture and the serum was analyzed by immunoblotting.

### Immunoblotting

Proteins separated by SDS-PAGE were transferred to an Immobilon-P transfer membrane (Merck Millipore, Billerica, MA, USA) and blocked with 1% (wt/vol) nonfat dry milk (WAKO, Osaka, Japan) in TBS-0.05% Tween 20 (TBS-T). The membranes were incubated overnight at 4°C with polyclonal antibody produced against live whole cells of *L. interrogans* serovar Manilae (anti-*L. interrogans* pAb) (1:1000 dilution) [57] or anti-HADH rabbit antiserum polyclonal antibody (1:5000), followed by incubation for 2 h at room temperature with horseradish peroxidase (HRP)-donkey anti-rabbit IgG conjugate (1:5000 dilution; GE Healthcare UK Ltd, Little Chalfont, UK), or with anti-His (C-term) mouse mAb (1:5000 dilution; Life technologies, Carlsbad, CA, USA), followed by incubation for 2 h at room temperature with horseradish peroxidase (HRP)-sheep anti-mouse IgG conjugate (1:10000 dilution; GE Healthcare UK Ltd, Little Chalfont, UK). The bands were detected with EzWest Lumi plus (ATTO, Tokyo, Japan) and ImageQuant LAS 4000mini (GE Healthcare UK Ltd, Little Chalfont, UK).

### Liquid chromatography (LC)/mass spectrometry (MS) analysis

Protein spots in gels were compared and analyzed by visual inspection. The gel spots were stored in 1% acetic acid and were subjected to LC/MS/MS analysis. Identification of proteins was carried out using Mascot server (Matrix Science) with datasets of rodent and *Leptospira* proteomes. A protein score of >40 was used to select proteins with significant matching. The difference between the theoretical and experimental mass and pI was also used to determine significant matching.

## Abbreviations

HADH: 3-hydroxyacyl-CoA dehydrogenase; CBB: Coomassie brilliant blue; ELISA: Enzyme-linked immunosorbent assay; HRP: Horseradish peroxidase; IEF: Isoelectric focusing; IPTG: Isopropyl-beta-D-thiogalactopyranoside; JICA: Japan International Cooperation Agency; JST: Japan Science and Technology Agency; LC: Liquid chromatography; MS: Mass spectrometry; MAT: Microscopic agglutination test; anti-L. interrogans pAb: Polyclonal antibody produced against live whole cells of *L. interrogans* serovar Manilae; PCR: Polymerase chain reaction; SATREPS: Science and Technology Research Partnership for Sustainable Development; SDS-PAGE: Sodium dodecyl sulfide–polyacrylamide gel electrophoresis; TBS-T: TBS-0.05% Tween 20; 2-DE: Two dimensional electrophoresis.

## Competing interests

The authors declare that they have no competing interests.

## Authors’ contributions

TS designed portions of the study, carried out all the experiments, and drafted the manuscript. KHN designed portions of the study, participated in the immunoassay, and revised the manuscript. SYAMV participated in the immunoassay and revised the manuscript. MS designed portions of the study and revised the manuscript. KN participated in the proteomic analysis and revised the manuscript. NGG participated in the design of the study. SY conceived and designed portions of the study, and revised the manuscript. All authors read and approved the final manuscript.

## Supplementary Material

Additional file 1: Table S1 Amino acid sequence coverage of leptospiral HADH by LC/MS/MS. Click here for file
