# Supplementary material for: Identification of leptospiral 3-hydroxyacyl-CoA dehydrogenase released in the urine of infected hamsters
Source: BMC Microbiol. 2014 May 21;14:132. doi: 10.1186/1471-2180-14-132 (PMC4036750; doi:10.1186/1471-2180-14-132)
Supplement: Additional file 1: Table S1 — Amino acid sequence coverage of leptospiral HADH by LC/MS/MS. [file 1471-2180-14-132-S1.doc]

**Additional file 1: Table S1 Amino acid sequence coverage of leptospiral HADH by LC/MS/MS.**

| **1** | MREIKTVTVL | GANGTMGAGS | AAIVASFGK**A** | **KVHMLARDTN** | **KAK**EGIEKAI |
| --- | --- | --- | --- | --- | --- |
| **51** | GSVKTDTIRP | RLIPGSYDAD | LEKAVSESDW | VFELVAESYE | VKEPINKRIA |
| **101** | SSRRPGTIVS | TVSSGLSIER | LSKAFDEDGQ | KHYFGTHFFN | PPYKMILCEL |
| **151** | VSHK**GSDKK**V | LKQLGEYLEK | VLGR**AVVYTN** | **DTPAFAGNR**I | GFQLINEVAQ |
| **201** | IAEKYSDKGG | IALMDAIMSG | YTGR**AMAPLD** | **TADFVGLDVH** | **K**AIVDNLYEM |
| **251** | TKDAAHSTFK | MPDYFQKLID | KGDLGRKTGG | GLYKMSKTPD | GKKEKLVYNI |
| **301** | GADLYEPVPK | FEIDFIRQAN | KR**ISEADYTG** | **AMNIVK**EAKG | FEADLARYFI |
| **351** | ARYVSYSLSI | VGEVVDTK**EM** | **ADLAMGTGFN** | **WAPASAFVDF** | **LGGPKDAIQL** |
| **401** | **IEK**AK**LPVPE** | **VLAKAKPGKP** | **FYELK**EKLDA | RSLFKG |  |
| Matched peptides are shown in bold. | | | | | |
